# Same-Day Counseling: Study Protocol for the Evaluation of a New Mental Health Service

**DOI:** 10.2196/resprot.5206

**Published:** 2016-02-03

**Authors:** Victoria Ewen, Aislin R Mushquash, Kathleen Bailey, John M Haggarty, Sumeet Dama, Christopher J Mushquash

**Affiliations:** ^1^ Lakehead University Thunder Bay, ON Canada; ^2^ St Joseph's Care Group Thunder Bay, ON Canada; ^3^ Northern Ontario School of Medicine Thunder Bay, ON Canada

**Keywords:** single-session counseling, mental health services, outpatient, program evaluation, access

## Abstract

**Background:**

Single-session counseling is being implemented across Canada to increase the accessibility and availability of mental health services. Despite increasing use, existing research on single-session counseling is sparse and has methodological limitations. In addition, some stakeholders are skeptical that this model of care can support meaningful change for clients.

**Objective:**

The aim of this study is to evaluate a new single-session counseling program (called Same-Day Counseling) offered in an outpatient community mental health clinic in Northwestern Ontario, Canada.

**Methods:**

Clients who attend Same-Day Counseling services will be given the opportunity to participate in the program evaluation. Those who consent will complete measures before their session, after their session, and at 1-month follow-up. Data will provide information on who accesses Same-Day Counseling (eg, typical presenting problems, symptom severity), client satisfaction with services, and whether clients benefit from the services (eg, improved functioning and reduced symptom severity).

**Results:**

Data collection is underway with 80 participants having completed baseline measures and 55 participants having completed follow-up measures. Data collection is expected to conclude in December 2015.

**Conclusions:**

This study is designed to contribute to the literature regarding the integration of single-session counseling into ongoing mental health services, with additional attention to methodological rigour. Our approach will help to address ongoing concerns regarding the implementation of single-session counseling, and inform health care providers and policy makers regarding the utility of this model for addressing the mental health care need of the community.

## Introduction

### Current State of Mental Health Services

Many Canadians are in need of mental health services, with these needs often going unmet, especially in the public system [1]. Service providers and administrators are acutely aware of the demand, yet struggle with limited resources available to provide the necessary care. As a result, mental health service agencies in Canada have long wait times (eg, up to 2 years) with some programs forced to temporarily shut their doors due to an inability to meet increasing demands [2-4]. This situation can result in deterioration of clients’ mental health conditions and increased reliance on acute medical care services (eg, emergency departments) [5]. Novel approaches to meeting clients’ needs are required to improve the accessibility and the availability of mental health services given the current resource limitations.

Once clients are registered with mental health services, high rates of missed appointments (eg, 15%) and dropouts (eg, 20%) are common and are related to reduced treatment outcomes [6,7]. For counselors, nonattendance initially results in underutilization of their time [6,7], as the missed session could have been made available to other clients. Afterwards, additional time is spent contacting clients who did not attend, which further restricts counselors’ time to see clients [6]. Innovative service solutions that address nonattendance issues are necessary in order to increase the efficiency of mental health services.

Difficulty determining treatment sufficiency in counseling also contributes to service inaccessibility. It is often unclear to counselors whether termination of ongoing care is appropriate [8,9]. Research into effective psychotherapy duration traditionally suggested a linear relationship between number of sessions and treatment outcomes, where a greater amount of counseling results in greater improvements [10]. Recent research, however, demonstrates that while longer durations of counseling are more effective overall, rapid improvement occurs early in treatment, with each additional session producing less significant results [11,12]. In fact, if clients have not experienced functional changes by the eighth session, the likelihood of significant change is greatly diminished [11]. This more recent understanding of rates of improvement throughout the course of counseling further highlights the need for novel approaches to service provision.

### Single-Session Counseling

One model of care, single-session counseling, has been implemented in an attempt to increase the availability and accessibility of mental health services. This type of counseling is broadly defined as any therapeutic encounter determined at the outset to be self-contained by both the counselor and the client [13]. The session is approached as a single encounter, regardless of the client’s intention to access the service in the future. Single-session counseling has been offered by mental health professionals through community mental health centers and counseling centers in Canada, the United States, Europe, and Australia, and is also available in primary care and educational settings [8]. It is predominantly utilized in the form of walk-in counseling, however, some programs give clients the opportunity to schedule an appointment on the day they would like to attend [8]. With most single-session counseling programs, the option to return for future sessions is presented [8]. Clients who access single-session counseling multiple times may or may not meet with the same counselor, as many programs employ a team approach [3,4]. Screening for more severe presenting problems, such as suicidal or homicidal ideation, occurs frequently within this model of care with the hope that these clients will be directed to alternative crisis management services.

Single-session counseling is often associated with a solution-focused or client-centered approach to service delivery [14,15]. In this form of counseling, clients are in charge of the frequency in which they seek out support from a counselor, thereby being more client-driven and increasing sense of control for the client. As a result, the single-session counselor is permitted to be resourceful and flexible in using a variety of counseling modalities to best meet the needs of the clients in the moment [14,15]. While single-session counselors report having a greater sense of urgency to clarify priorities during each contact [15], they vary in their approaches to accomplishing this goal.

Different procedures are utilized to integrate single-session counseling into currently existing mental health services. One option is to replace the intake appointment with single-session counseling [4]. Single-session counseling can also be provided to clients that are on the waitlist for future services [16]. Other programs operate as stand-alone services with no direct connection to additional programming. Flexibility in the implementation of single-session counseling, and incorporation into ongoing mental health services, has resulted in an increase in this model of care in Ontario, Canada [3].

Integration of single-session counseling into current services has the potential to address many issues faced by mental health service providers. There is little to no wait for services with single-session counseling, allowing clients to access services when motivation and need are highest. Dropouts do not exist in single-session counseling, as there is no expectation of ongoing sessions. The self-contained format, along with the short-term nature of the scheduling process, also greatly reduces rates of missed appointments. Finally, treatment sufficiency is no longer an issue, as each session of counseling is considered sufficient, regardless of whether the client returns for future sessions [8,9]. Due to these factors, the single-session counseling format presents a possible option for meeting the current needs of clients, mental health care providers, and program administrators.

### Available Evidence on Single-Session Counseling

Although research is limited, there is preliminary evidence for the utility of single-session counseling in reducing barriers to services, increasing client satisfaction, and helping clients address their mental health concerns. A review conducted by Hymmen et al [8], found anywhere from 74% to 100% of clients are satisfied across a wide range of single-session services. Also, 61% of clients found a single session of counseling sufficient to meet their needs and that they did not require additional sessions [8]. Single-session counseling is associated with symptom reduction and improvement in coping with presenting problems [8,9]. Receiving helpful advice about the problem, having the opportunity to talk about the problem and feel supported, and the immediate accessibility of mental health services were identified as helpful aspects of single-session counseling [8]. Findings are mixed regarding the relationship between type and severity of the presenting problem and outcomes in single-session counseling [8].

Despite these promising results, additional evidence is needed. Moreover, the available research has methodological issues that reduce the ability to draw firm conclusions about the utility of single-session counseling. For example, most services lack ongoing standardized outcome measurement for evaluation, relying mainly on client satisfaction questionnaires, and/or employee-created measures that lack psychometric data on their reliability and validity [8]. Another limitation is the involvement of the counselors in data collection and analysis, which may lead to response bias among participants [8]. Evaluations have also exhibited high attrition rates (eg, upwards of 50%) [17], which may be attributable to the method of data collection [3]. Lastly, restrictions on who can access services (eg, limited to clients without suicidal or homicidal ideation) reduce the generalizability of the results to a larger client base [3,8]. These limitations result in uncertainty regarding the utility of this service model and further emphasize the need for additional research.

In addition to the limitations of existing evidence, some counselors and decision makers have reservations about brief interventions [4,18], believing that they do not address the underlying problem [19]. Some counselors concede that single-session counseling can be effective, however, view it as ineffective or even ill-advised for clients with more complex or severe presenting problems [8,14]. Additional concerns exist that the increased presence of single-session counseling programs is due to demands placed on health service providers and is not in the best interest of the clients [19]. Further evidence is necessary to support the use of single-session counseling and validate its integration into mental health services.

### Aims and Hypotheses

**Figure 1:**
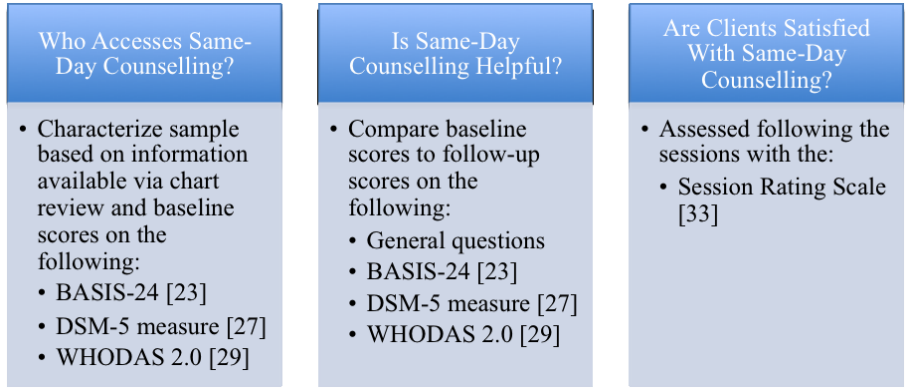
Summary of evaluation questions, domains assessed, and outcome measures included.

This paper describes the protocol our team will use to evaluate a new single-session counseling program (called Same-Day Counseling) offered in an outpatient community mental health clinic in Northwestern Ontario, Canada. Our study was designed with increased attention to methodological concerns including the use of multiple standardized measures, exclusion of the counselor from data collection and analysis, use of phone calls for follow-up evaluation to reduce attrition from the evaluation, and a lack of restrictions on who can access the program. Participants will be characterized in terms of demographic variables, symptomatology, contact with mental health services, and wait times. In addition, this evaluation will examine not only client satisfaction, but also changes in scores on measures of mental health functioning, psychiatric symptoms, and general health functioning. It is hypothesized that participants will be satisfied with the service, and their ability to manage their presenting problems as well as their mental health, general health and functioning will increase, and their symptoms will decrease. The evaluation questions, along with the corresponding measures and domains being assessed, are presented in Figure 1.

## Methods

### Evaluation of a New Program

Our study will evaluate a novel single-session counseling service, referred to as Same-Day Counseling, using a single group pre-post design. Same-Day Counseling adheres to the single-session service model, in that each session is considered self-contained. The Same-Day Counseling program is provided in a prescheduled format in which clients book an appointment for the day they would like to attend by calling reception staff that morning and selecting an available time that day (ie, they call in on the “same-day” they would like to attend). Clients are able to access the service as often as they would like, and have the option of meeting with the same counselor should they attend multiple sessions. There are no restrictions on who can access the service in terms of symptom severity or presenting problem.

### Setting

The program evaluation will take place at a large outpatient community mental health clinic in Thunder Bay, Ontario, Canada (ie, St Joseph's Care Group Mental Health Outpatient Programs). Thunder Bay is a city in Northwestern Ontario that has a population of about 108,000 and operates as a hub for health care in the region [20,21]. Although rurally located, this city contains a regional hospital, an international airport, as well as a college and a university.

The clinic where the evaluation will take place serves adults with serious and persistent mental health problems and promotes the principles of psychosocial rehabilitation and recovery within an evidence-based and best practices model of care [22]. Services offered throughout the program include assessment, individual counseling, group counseling, medication management, spiritual care, referrals to community resources, advocacy, and primary health care as required [22]. Self-referrals and referrals from other service providers (eg, family physicians, nurse practitioners) are accepted. Services are publically funded through the Ontario government, and provided at no cost to the clients upon presentation of an Ontario Health Insurance Plan card.

### Participants

Clients registering for outpatient mental health services who are not yet connected to an individual counselor will be provided with information regarding the Same-Day Counseling program. Those who pursue Same-Day Counseling will be informed of the evaluation and invited to participate. There are no exclusion criteria. For participating in the evaluation, clients will be compensated with a US $10 gift certificate to a local coffee shop and entered into one of 5 draws for a US $100 grocery gift certificate.

### Intervention

Same-Day Counseling sessions are typically 60-90 minutes in length and focus on addressing clients’ immediate mental health concerns. Counselors are registered social workers with previous experience providing counseling within the clinic. Incoming counselors are given a general overview of the model and the rationale behind the service. No formal training was completed regarding the provision of Same-Day Counseling, as they are expected to use their clinical judgment and evidence-based interventions as they would with traditional forms of therapy. In general, interventions delivered include techniques from cognitive-behavioral therapy, dialectical behavior therapy, and emotion-focused therapies, among others. Counselors also assist clients with functional tasks associated with goal setting, securing safe housing, navigating legal matters, and promoting health and wellness. Counselors do not book follow-up appointments with clients, but welcome clients to attend Same-Day Counseling in the future, as needed.

### Outcome Measures

The research team reviewed relevant literature and consulted with the counselors when choosing the outcome measures. Considerations included breadth and depth, length, clinical utility, accessibility, as well as their psychometric properties. Researchers generated 4 general questions (see Multimedia Appendix 1) that will be used to assess participants’ ability to manage their presenting problems including the amount of stress the problem is causing, the amount of understanding participants have related to the cause of the problem, the amount of confidence participants have to cope with the problem, and the amount of knowledge, supports, or resources participants have to manage the problem.

The Behavior and Symptom Identification Scale-24 (BASIS-24) [23] will be included as a measure of mental health symptoms and functioning. This measure provides an overall score as well as subscale scores for the following domains: depression, interpersonal relationships, psychotic symptoms, alcohol/drug use, emotional lability, and self-harm [24]. The BASIS-24 displays excellent psychometric properties in outpatient samples (eg, internal consistency alpha =.70-.81; concurrent validity *r*=.59-.82) [25,26].

The Diagnostic and Statistical Manual-5 Level 1 Cross Cutting Symptom Measure for Adults (DSM-5 Measure) will be used to assess a variety of domains of psychiatric symptom frequency and intensity, including depression, anger, mania, anxiety, somatic symptoms, suicidal ideation, psychosis, sleep problems, memory, repetitive thoughts and behaviors, dissociation, personality functioning, and substance use [27]. The DSM Measure is a reliable instrument in North American populations (eg, test re-test reliability *r*=.66-.97) [28].

The World Health Organization Disability Assessment Schedule 2.0 (WHODAS 2.0) will be included as a measure of general health, functioning, and impairment [29]. The following six domains of functioning are assessed: cognition, mobility, self-care, getting along, life activities, and participation in society [30]. The WHODAS 2.0 has been used worldwide, and is psychometrically validated for use among individuals with common mental health disorders (eg, internal consistency alpha=.89; convergent validity *r*=.58) [31,32].

Finally, the Session Rating Scale (SRS) will be used as a measure of client satisfaction. The SRS is a brief global measure of therapeutic alliance designed to be easily administered in a clinical setting [33], while maintaining reliability and validity for individuals accessing outpatient mental health services (eg, internal consistency alpha=.88; convergent validity *r*=.48; test re-test reliability *r*=.64-.70) [34].

### Procedure

Ethics approval for the study was obtained through a hospital research ethics board (ie, St Joseph’s Care Group) and a university research ethics board (ie, Lakehead University). All participants will be informed that participation, nonparticipation, or withdrawal from the evaluation will not affect the care received. Documentation of participants’ involvement in the study will not be included in their clinical chart, and counselors will not be involved in recruitment, data collection, or data analysis. To maintain confidentiality, personal identifying data will be stored separately from evaluation information. Only the researcher team will be able to access both files.

Clients attending Same-Day Counseling who consent to participate in the evaluation will be given a questionnaire package by reception staff. Measures completed pre-session, post-session, and at 1-month follow-up are presented in Table 1.

**Table 1:** Timeline for administration of outcome measures.

Presession	Postsession	One-month follow-up
General questions	General questions	General questions
	Session Rating Scale (SRS)	
Behavior and Symptom Identification Scale-24 (BASIS-24)		BASIS-24
World Health Organization Disability Assessment Schedule 2.0 (WHODAS 2.0)		WHODAS 2.0
Diagnostic and Statistical Manual-5 Self-Rated Level 1 Cross Cutting Symptom Measure for Adults (DSM 5 Measure)		DSM 5 Measure

Following their session, participants will leave the completed questionnaire package with reception staff. Approximately 1 month after their session, participants will be contacted by phone by a research assistant to complete the follow-up measures. Also after the session, the research team will conduct a chart review to characterize the individuals accessing Same-Day Counseling in terms of demographic and service utilization information.

If a participant endorses suicidality on the evaluation questionnaire, he or she will be informed by a research assistant that his or her counselor will be in touch to assess the current level of risk and to provide immediate support. Crisis response contact information will also be provided. A counselor will attempt to contact the participant by phone within 24 hours to further assess the risk. This protocol is put in place to keep clients safe and to ensure that researchers do not enter into dual relationships with the clients (eg, researcher and counselor).

### Statistical Analyses

Descriptive statistics will be conducted for participants in the Same-Day Counseling program to quantify demographic variables, symptomatology, contact with mental health services, and wait times, as well as participants’ ratings of the counseling session. Paired-samples *t* tests will be used to compare pre-session and follow-up scores on participants’ ability to manage the presenting problem, their psychiatric symptom frequency and intensity, their mental health and functioning, and their general health and functioning.

### Sample Size Calculation

A power analysis was conducted using G*Power Software to determine the required sample size. Given a 2-sided alpha level of .01, a power of .8, and an intended effect size of .56, the required sample size is 41 participants. The chosen effect size was informed by research on the psychometric properties of the BASIS-24 [24]. Rates of attrition in single-session counseling service evaluations vary greatly depending on the type of follow-up contact and the duration between baseline and follow-up [8]. Based on an expected attrition rate of 30%, the current evaluation requires a sample size of 59 participants [35]. Participants will be recruited from the Same-Day Counseling program until sufficient sample size at follow-up has been achieved.

## Results

Participant recruitment began in February 2014. Currently, 80 participants consented and completed pre- and post-session measures; 18 of the participants completed the baseline questionnaires on multiple occasions, for a total of 110 completed baselines. So far, 55 participants have completed 1-month follow-up measures. Data collection is expected to conclude in December 2015, with an estimated 76 participants, based on the current rate of attrition.

## Discussion

Single-session counseling is a potential option for meeting the needs of clients, one that has been available throughout Canada for a number of years. Program evaluations of single-session counseling services provide preliminary evidence supporting its use, but often lack attention to methodology. Additional research is required to ensure that the continued implementation of this model of care is substantiated by evidence.

The results of this study will contribute to the research on integration of single-session counseling into ongoing mental health services, and hopefully encourage further research into such programs. This evaluation will also attempt to address the ongoing concerns that implementation of single-session counseling may be motivated by factors other than the best interest of clients. Ultimately, additional literature on single-session counseling would help inform service providers and policy makers surrounding the best use of mental health service resources.

## Appendix

Multimedia Appendix 1 General Questions.

Multimedia Appendix 2 Funding approval.

## Abbreviations

BASIS-24: Behavior and Symptom Identification Scale-24
DSM 5 Measure: Diagnostic and Statistical Manual-5 Level 1 Cross Cutting Symptom Measure for Adults
SRS: Session Rating Scale
WHODAS 2.0: World Health Organization Disability Assessment Schedule 2.0
