# Supplementary material for: Same-Day Counseling: Study Protocol for the Evaluation of a New Mental Health Service
Source: JMIR Res Protoc. 2016 Feb 3;5(1):e22. doi: 10.2196/resprot.5206 (PMC4757779; doi:10.2196/resprot.5206)
Supplement: Multimedia Appendix 1 [file resprot_v5i1e22_app1.pdf]

Multimedia Appendix 1

General Questions

When you first arrived, you listed the main problem that brought you to single-session counselling. Please complete the following questions about that main problem by choosing a number from 1 (none) to 10 (extreme).

	None			A little				A lot		
1. How much stress is the problem causing you?	1	2	3	4	5	6	7	8	9	10
2. How much of an understanding do you have about what is causing the problem?	1	2	3	4	5	6	7	8	9	10
3. How much confidence do you have to fix, reduce, or cope with the problem?	1	2	3	4	5	6	7	8	9	10
4. How much do you know about finding supports or resources to help fix, reduce, or cope with the problem?	1	2	3	4	5	6	7	8	9	10
