# Supplementary material for: Same-Day Counseling: Study Protocol for the Evaluation of a New Mental Health Service
Source: JMIR Res Protoc. 2016 Feb 3;5(1):e22. doi: 10.2196/resprot.5206 (PMC4757779; doi:10.2196/resprot.5206)
Supplement: Multimedia Appendix 2 [file resprot_v5i1e22_app2.pdf]

NOAMA

NORTHERN ONTARIO ACADEMIC MEDICINE ASSOCIATION

April 14, 2014

Dr. John Haggarty, Professor
NOSM Clinical Sciences Division

Dear Dr. Haggarty:

The Northern Ontario Academic Medicine Association (NOAMA) is pleased to inform you that your application for Clinical Innovation Opportunities funding has been approved by the NOAMA Board of Directors.

Your project entitled **"A Pilot Evaluation of a Single-Session Counselling Service for Patients Presenting to St. Joseph's Care Group Mental Health Outpatient Programs"** has received approval for the following funding:

| Year 1 | Year 2 | Total |
|---|---|---|
| $49,910 | - | $49,910 |

The approved funds will be released to an appropriate transfer agency designated by you on your Project Submission Form, in accordance with the **Clinical Innovation Opportunities Fund Guidelines and Application Process** summarized in the attached **Process for the Distribution of Funds** document.

All publications arising from the Clinical Innovation Opportunities Fund are to acknowledge *"Supported by the Northern Ontario Academic Medicine Association (NOAMA) Clinical Innovation Opportunities Fund award"* and are to include the NOAMA logo. The logo is available from the NOAMA office.

If you have any questions or require further information, please contact the NOAMA office by email at noama@noama.ca or by phone at (807) 766-7357.

We look forward to the results of the successful completion of your innovation project. Northern physicians have great ideas!

Sincerely,

Dr. Rayudu Koka
Co-Chair, NOAMA Board

Dr. David Marsh
Co-Chair, NOAMA Board

Attachments (1)

c.c. Dr. William McCready, Chair
Clinical Innovation Opportunities Fund Sub-Committee
